# Brusatol Inhibits Tumor Growth and Increases the Efficacy of Cabergoline against Pituitary Adenomas

**DOI:** 10.1155/2021/6696015

**Published:** 2021-06-16

**Authors:** Zerui Wu, Yunqiu Xu, Jiadong Xu, Jianglong Lu, Lin Cai, Qun Li, Chengde Wang, Zhipeng Su

**Affiliations:** ^1^Department of Neurosurgery, First Affiliated Hospital of Wenzhou Medical University, Wenzhou 325000, China; ^2^Department of Cardiothoracic Surgery, Zhoushan Hospital, Wenzhou Medical University, Zhoushan 316000, China

## Abstract

Cabergoline (CAB) is the first choice for treatment of prolactinoma and the most common subtype of pituitary adenoma. However, drug resistance and lack of effectiveness in other pituitary tumor types remain clinical challenges to this treatment. Brusatol (BT) is known to inhibit cell growth and promote apoptosis in a variety of cancer cells. In our present studies, we investigate the effects of BT on pituitary tumor cell proliferation *in vitro* and i*n vivo*. BT treatment resulted in an increase in Annexin V-expressing cells and promoted the expression of apoptosis-related proteins in rat and human pituitary tumor cells. Investigation of the mechanism underlying this effect revealed that BT increased the production of reactive oxygen species (ROS) and inhibited the phosphorylation of 4EBP1 and S6K1. Furthermore, treatment with a combination of BT and CAB resulted in greater antitumor effects than either treatment alone in nude mice and pituitary tumor cells. Collectively, our results suggest that the BT-induced ROS accumulation and inhibition of mTORC1 signaling pathway leads to inhibition of tumor growth. Combined use of CAB and BT may increase the clinical effectiveness of treatment for human pituitary adenomas.

## 1. Introduction

Brusatol (BT) is a natural product obtained from *Brucea javanica*, a common evergreen shrub used in traditional Chinese medicine [1]. BT exerts an array of biological effects, including triggering anticancer and anti-inflammatory activity [2, 3]. The mechanism underlying the anticancer activity of BT involves its effects on protein synthesis and ROS [4]. For example, Mata-Greenwood et al. observed that BT exhibits cytotoxic effects on leukemic cells via inhibition of c-Myc expression [5]. In addition, BT increases the efficacy of chemotherapy by inhibiting NRF2 expression in lung cancer cells [6] and suppresses glioma tumor growth by interrupting glutathione metabolism [7]. BT is also reported to induce autophagy and apoptosis in hepatocellular carcinoma via inhibition of the PI3K/AKT/mTOR pathway [8]. Whether BT has antitumor effects on pituitary tumors is unknown.

Pituitary adenoma is one of most common intracranial tumors, with prolactinoma accounting for ~45% of these tumors [9, 10]. Cabergoline, a dopamine type 2 receptor (DRD2) agonist, is the first-choice treatment [11] for prolactinomas because its ability to reduce prolactin secretion results in decreased tumor volume in most patients [12]. However, 10%–20% of prolactinomas fail to respond to cabergoline therapy, which means that these patients were resistant to cabergoline therapy [13, 14]. The protein kinase mTOR is a major component of the mTORC1 and mTORC2 complexes [15] that play a pivotal role in tumor progression and responses to chemical therapy [16]. DEPTOR, an important modulator of mTOR [17], is downregulated in pituitary adenomas. DEPTOR expression in pituitary tumor GH3 and MMQ cells was observed to inhibit their proliferation and to increase their sensitivity to cabergoline *in vitro* and *in vivo* via inhibition of mTORC1 [18]. Guo et al. observed that BT exerts anticancer effects in nasopharyngeal carcinoma via suppression of the AKT/mTOR signaling pathway and inhibits cell proliferation by inhibiting the PI3K/AKT/mTOR pathway [8, 19]. Our previous studies have shown that cabergoline impairs autophagic flux and induces autophagic cell death by inhibiting the AKT/mTOR pathway [20]. Collectively, these results suggest that BT may inhibit the mTOR pathway in pituitary adenomas. This study is aimed at determining whether BT exerts antitumor effects on pituitary adenoma and whether it acts synergistically with cabergoline to solve the problem of drug resistance.

## 2. Materials and Methods

### 2.1. Cell Lines and Culture Conditions

Rat pituitary tumor GH3 cell lines (ATCC CRL-10609) and MMQ cell lines (ATCC CRL-10609) were purchased from the American Type Culture Collection (ATCC). These cells were cultured in a 5% CO_2_ atmosphere at 37°C. Cells were cultured in Ham's F-12K (Kaighn's) medium (Gibco, Life Technologies) supplemented with 12.5% horse serum (Gibco), 2.5% fetal bovine serum (Gibco), and 1% penicillin/streptomycin (Gibco). Cabergoline and BT were purchased from Tocris Bioscience (Cat. No. 2664, UK) and MedChemExpress (HY-19543, USA).

### 2.2. Cell Viability and Apoptosis Assays

GH3 and MMQ cells were seeded in 96-well cell culture plates (5 × 10^3^ cells/well) and treated with appropriate doses of each drug for 24 h and 48 h. Cell survival was assayed using the MTS (3-(4,5-dimethylthiazol-2-yl)-5-(3-carboxymethoxyphenyl)-2-(4-sulfophenyl)-2H-tetrazolium, inner salt)-based solution cell proliferation assay kit (Promega, G3580) according to the manufacturer's instructions. After adding the MTS solution, the reaction plate was incubated at 37°C for 1–2 h, and the absorbance was read at 490 nm with a plate reader (TECAN, Switzerland). The percentage of surviving cells was calculated using the following formula: [(*A*_490_ sample − *A*_490_ background)/(*A*_490_ control − *A*_490_ background)] × 100%. For the apoptosis test, cells were stained with Annexin V-PI as described by the manufacturer (BD Biosciences, Cat#556547) and assayed by flow cytometry (CyAn ADP, Beckman Coulter, Brea, CA, USA).

### 2.3. Colony Formation Assays

GH3 and MMQ cells (1 × 10^3^ cells/well) were plated into 6-well plates and treated with BT and cabergoline or phosphate-buffered saline for 14 days. After fixation with 4% paraformaldehyde, cells were stained with 1% crystal violet staining solution (Sangon Biotech, E607309–0100). After extensive washing and air drying, the plates were photographed using a digital camera. Cell colonies were counted using PhotoShop CS6.

### 2.4. Prolactin and Growth Hormone Assay via ELISA

MMQ and GH3 cells (5 × 10^5^ cells/well) were plated into 6-well plates and treated with a range of doses of BT (0, 50, 100, 200, and 500 nM) for 24 h followed by harvesting of the cell supernatant. The concentrations of prolactin (Catalog#HLE20788, Haling Biotechnology Co., Ltd., Shanghai, China) and growth hormone (GH, Catalog#HLE20670, Haling Biotechnology Co., Ltd., Shanghai, China) in supernatant were determined by using ELISA assay kits. Experiments were carried out according to the manufacturer's instructions. The absorbance at 490 nm was acquired measured using a VersaMax Tunable MicroPlate Reader (RT-6100, Rayto, China).

### 2.5. Western Blot Analysis

After being treated with appropriate doses of each drug for indicated time, cell samples, including GH3, MMQ, and primary pituitary tumor cells, were harvested in sterile Eppendorf tubes and washed with cold PBS for two times. Then, these samples were lysed in lysis buffer (50 mM Tris [pH 7.5], 120 mM NaCl, and 0.5% NP-40) containing protease and phosphatase inhibitors and placed on ice for 30 minutes. After that, they were centrifuged at 12,000 × g for 10 minutes. The total protein concentration of the samples was measured using the bicinchoninic acid protein assay kit (Tiangen Biotech, PA115). The protein samples were separated by SDS-PAGE and immunoblotted with the indicated antibodies. The LAS4000 system was used for imaging, and protein band intensity was quantified by densitometry using the ImageJ software. The following antibodies were used in this study: Tubulin antibody (11224-1-AP; Proteintech Group), S6K1 (Cat#2708, Cell Signaling Technology), p-S6K1-Thr389 (Cat#9234, Cell Signaling Technology), 4EBP1 (Cat#9644, Cell Signaling Technology), p-4EBP1 (Cat#2855, Cell Signaling Technology), Antirabbit IgG, HRP-linked antibody and Anti-mouse IgG, and HRP-linked antibody (#7076; #7074, Cell Signaling Technology).

### 2.6. Xenograft Tumor Model

All procedures were carried out according to the Institutional Animal Care and Use Committee protocol approved for this study by Wenzhou Medical University and performed in accordance with the National Institutes of Health Guide for the Care and Use of Laboratory Animals. Female nude mice (BALB/c-nu) were purchased from the SLAC (Shanghai Slack Laboratory Animal Co., Ltd., Shanghai, China), which were kept under specific pathogen-free conditions. A total of 5 × 10^6^ of the indicated MMQ and GH3 cells were resuspended in 100 *μ*L PBS buffer mixed with Matrigel (1 : 1; BD Biosciences, USA, #356234) and then subcutaneously injected into the back of each nude mice. When the tumors reached an average size of 50 mm^3^, the mice were randomized into four groups, control group, group of BT treatment, group of CAB treatment, and group with BT combined with CAB treatment (MMQ xenograft models were only randomized into control group and BT-treated group). Subcutaneous local tumors were measured on length and width by a Vernier caliper every 2 days. Tumor volumes were calculated individually using the formula (length × width^2^)/2. At the end of the experiment, all mice were anesthetized and sacrificed, and tumors were harvested, followed by immunohistochemical staring analysis.

### 2.7. Immunohistochemistry Analysis

Xenograft tumor samples were fixed in 10% neutral buffered formalin for 24 hours at room temperature and dehydrated and embedded in paraffin as previously reported [20]. Paraffin-embedded tumor tissue sections were boiled in sodium citrate buffer (pH 6.0) for 30 min to retrieve the antigens. The sections were dehydrated and blocked with peroxidase treatment. For immunohistochemical staining, tissue sections were incubated with p-S6K1 antibody (ab60948, Abcam, USA) and p-4EBP1 (#2855, Cell Signaling Technology) overnight at 4°C followed by incubation with goat anti-mouse horseradish peroxidase secondary antibody (ab6788, Abcam; 1: 200 in 1% BSA/TBST) for 1 h at room temperature. The sections were then exposed to DAB substrate (dissolved in Dako substrate buffer; Roche, 760–500), followed by Gill's Hematoxylin counterstaining (Sangon Biotech, E607317) and standard dehydration treatment. Images of the stained sections were obtained using an axiovert microscope.

### 2.8. ROS Determination

According to the reference [21], BT-treated GH3 cells were washed once with warm PBS and then incubated with 10 *μ*M 2′,7′-dichlorodihydrofluorescein diacetate (DCFH-DA, Beyotime, S0033) in serum-deprived F12K medium. After incubating in the dark for 20 min at 37°C, the cells were incubated for an additional 10–15 min. Then, GH3 cells were harvested with 0.05% trypsin-EDTA solution, washed once with warm PBS, and analyzed by flow cytometry (CyAn ADP, Beckman Coulter, Brea, CA, USA). BT-treated MMQ cells were washed once with warm PBS and then incubated with 10 *μ*M DCFH-DA in serum-deprived F12K medium in the dark for 20 min at 37°C. Then, the cells were incubated for an additional 10–15 min and immediately analyzed with flow cytometer.

### 2.9. Primary Human Pituitary Tumor Cells

Our study was approved by the Clinical Research Ethics Committee of the First Affiliated Hospital of Wenzhou Medical University. Primary human pituitary tumor cells were obtained from patients who underwent surgeries for pituitary tumors between October 2019 and February 2020 at the Department of Neurosurgery, First Affiliated Hospital of Wenzhou Medical University, Wenzhou, China (Supplementary Table 1). The surgical samples were mechanically and enzymatically dispersed, and the primary tumor cells were carefully cultured in DMEM with 10% fetal bovine serum and 100 U/mL penicillin/streptomycin. For the MTS assay, primary human pituitary tumor cells were seeded in 96-well cell culture plates and treated with appropriate doses of brusatol for 24 h and then conducted cell activity experiments. For the Western blot analysis, primary tumor cells were seeded in a 6-well plate at a density of 1 × 10^6^ cells/well and treated with brusatol for 24 h. Pretreated primary cell samples were harvested and lysed in in lysis buffer for the next immunoblotting test.

### 2.10. Statistical Analysis

All experiments were performed using 5 mice or 3 independent repeated experiments with cells. Unless otherwise indicated, data are presented as the mean ± SEM. Student's unpaired, 2-tailed *t*-test with a 95% confidence interval was used to analyze data involving direct comparison of an experimental group with a control group. *P* < 0.05 was considered significant.

## 3. Results

### 3.1. BT Inhibits Pituitary Tumor Cell Proliferation and Growth In Vitro

**Figure 1:**
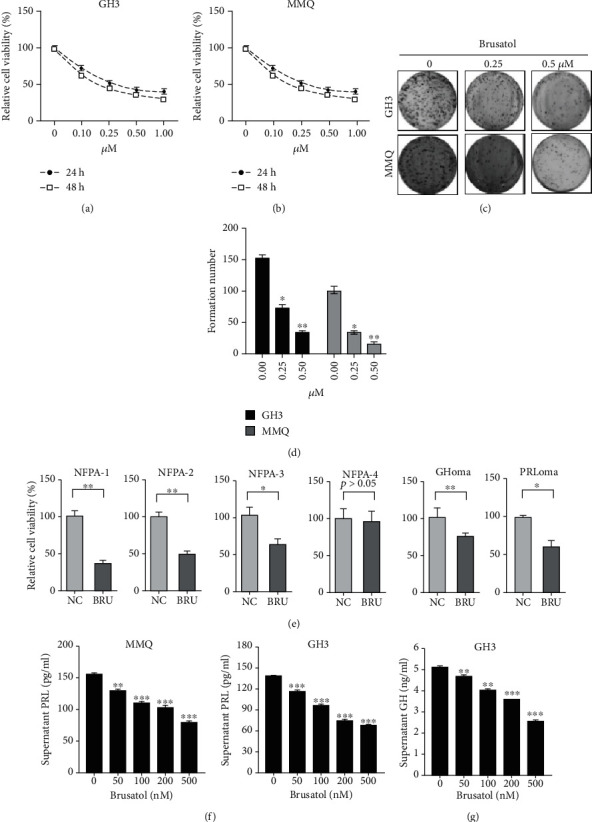
Brusatol (BT) repressed the growth and hormone secretion of pituitary adenomas. (a, b) GH3 and MMQ cells were treated with a range of concentrations of BT for 24 and 48 h. Cell viability was determined by MTS. (c, d) GH3 and MMQ cells were treated with BT for 24 h; formed colonies were photographed and counted using the ImageJ software. (e) Different subtypes of primary pituitary tumor cells were treated with 0.25 *μ*M BT for 24 h; cell viability was determined by MTS. (f) After treating GH3 and MMQ cells with BT (0–500 nM) for 24 h, culture medium supernatants were collected for ELISA to determine the prolactin hormone concentration. (g) After treating GH3 cells with BT (0–500 nM) for 24 h, culture medium supernatants were collected for ELISA to determine the levels of GH hormone. Data are represented as the mean ± SD. ^∗^*P* < 0.05; ^∗∗^*P* < 0.01; ^∗∗∗^*P* < 0.001.

To investigate the antitumor effects of BT on pituitary tumors, we incubated GH3 and MMQ cells with BT (0–1000 nM) for 24 h and 48 h and then assessed cell viability by MTS assay. BT caused a concentration-dependent decrease in the viability of GH3 and MMQ cells at 24 h and 48 h; treatment with BT at 250 nM significantly reduced GH3 and MMQ cell viability by approximately 50% (Figures 1(a) and 1(b)). Confirming these findings, colony formation assay showed that the numbers of colonies in GH3 and MMQ cells dramatically decreased after treatment with BT (Figures 1(c) and 1(d)). We investigated the effect of BT on *in vitro* growth of primary cultures of 6 human pituitary adenomas, including 4 nonfunctional tumors, 1 prolactin-secreting tumor, and 1 GH-secreting tumor. BT treatment at 250 nM decreased cell viability in 83.3% (5/6) of these tumors (Figure 1(e)).

Assessment of prolactin and growth hormone secretion from BT-treated GH3 and MMQ revealed that prolactin levels decreased in a dose-dependent manner in MMQ (control, 154.8 ± 2.08 pg/mL; 500 nM, 78.97 ± 3.80 pg/mL, *P* < 0.001) and GH3 cells (control, 134.4 ± 2.32 pg/mL; 500 nM, 67.77 ± 0.68 pg/mL, *P* < 0.001) (Figure 1(f)). GH secretion decreased significantly in GH3 cells treated with BT (control, 5.075 ± 0.21 ng/mL; 500 nM BT, 2.565 ± 0.07 ng/mL; *P* < 0.001) (Figure 1(g)). These data indicate that BT inhibited tumor growth and hormone secretion by pituitary adenomas *in vitro*.

### 3.2. BT Inhibits Tumor Growth of GH3 and MMQ Cells In Vivo

**Figure 2:**
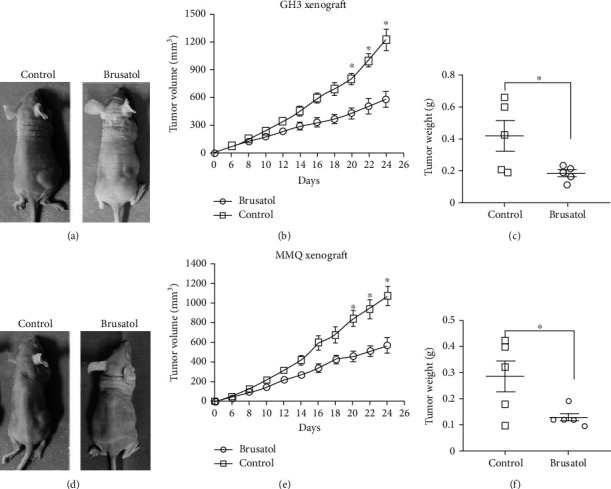
Brusatol (BT) inhibits pituitary tumor growth in xenograft models. (a, d) Representative images of xenograft tumors from mice treated with control vehicle or BT at 24 days of drug administration. (b, e) Tumor volume growth curves of nude mice in different treatment groups. BT inhibited the rate of tumor growth in GH3 and MMQ cells. (c, f) Tumor weights from mice injected with GH3 cells and treated with control vehicle or BT at day 24 of drug administration. Data are represented as the mean ± SD. ^∗^*P* < 0.05.

To evaluate the *in vivo* antitumor activity of BT, we established a xenograft model by subcutaneously inoculating nude mice with GH3 and MMQ cells. Representative images of mice harboring GH3 or MMQ xenografts at 24 days are shown in Figures 2(a) and 2(d). Tumors from the BT-treated group were significantly smaller than those from the control group (Figures 2(b) and 2(e)), as determined by tumor volume (GH3 cells: BT-treated, 576.3 ± 149.2 mm^3^; control, 1219.0 ± 200.2 mm^3^; *P* < 0.05; MMQ cells: BT-treated, 565.8 ± 139.8 mm^3^; control, 1069.0 ± 163.2 mm^3^; *P* < 0.05). The tumor weight was significantly decreased by BT treatment in GH3 (control, 0.42 ± 0.21 g; BT, 0.19 ± 0.04 g) and MMQ xenograft models (control, 0.28 ± 0.13 g; BT, 0.13 ± 0.03 g) (Figures 2(c) and 2(f)).

### 3.3. BT-Induced Significant Apoptotic Cell Death in GH3 and MMQ Cells

**Figure 3:**
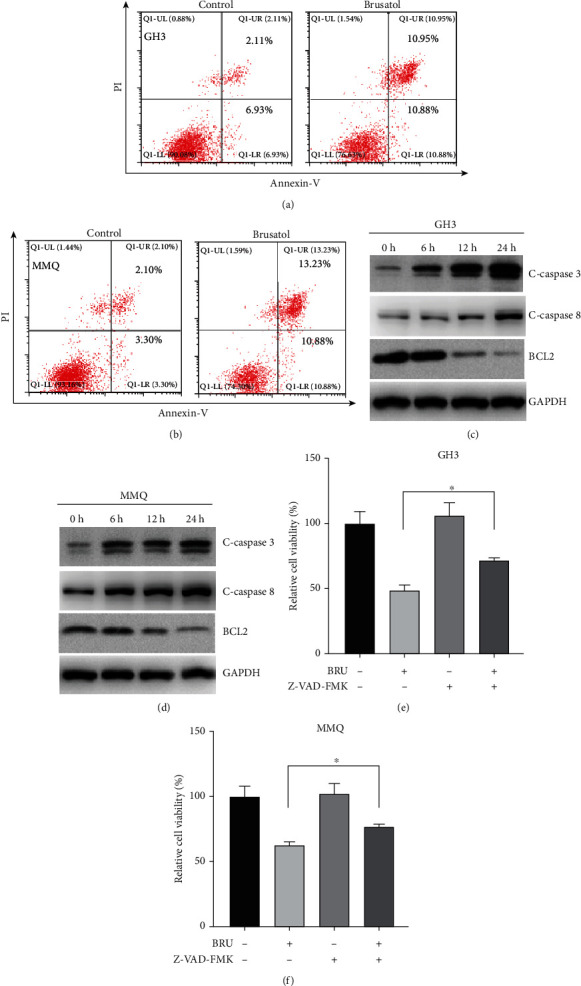
Brusatol- (BT-) induced apoptotic cell death in GH3 and MMQ cells. (a, b) Induction of apoptosis in GH3 and MMQ cells with BT treatment (250 nM) for 24 h followed by Annexin V and PI staining. Apoptosis ratios were measured by flow cytometry. (c, d) After 0–24 h treatment with BT (250 nM) in GH3 and MMQ cells, Western blot analysis was used to monitor the expression level of c-caspase-3, c-caspase-8, and Bcl-2. (e, f) GH3 and MMQ cells were treated with BT or NAC (100 *μ*M) alone or in combination for 24 h and MTS assays conducted to determine cell viability. Data are represented as the mean ± SD. ^∗^*P* < 0.05.

Apoptosis assays using PI and Annexin V-FITC double staining revealed that BT treatment induces apoptosis in GH3 and MMQ cells. BT increased the rate of apoptosis by 18.71% in MMQ cells and 12.79% in GH3 cells (Figures 3(a) and 3(b)). Western blot analysis showed that BT increased the expression of the apoptosis-related protein cleaved caspase-3 and -8 and downregulated Bcl-2 expression in GH3 and MMQ cells in a time-dependent manner (Figures 3(c) and 3(d)). The addition of the pan-caspase inhibitor Z-VAD-FMK blocked BT-induced cell death (Figures 3(e) and 3(f)). These data indicate that BT induces apoptotic cell death and exerts antitumor effects on pituitary tumors *in vitro* and *in vivo*.

### 3.4. BT Inhibits mTORC1 Pathway Activation in Pituitary Tumors

**Figure 4:**
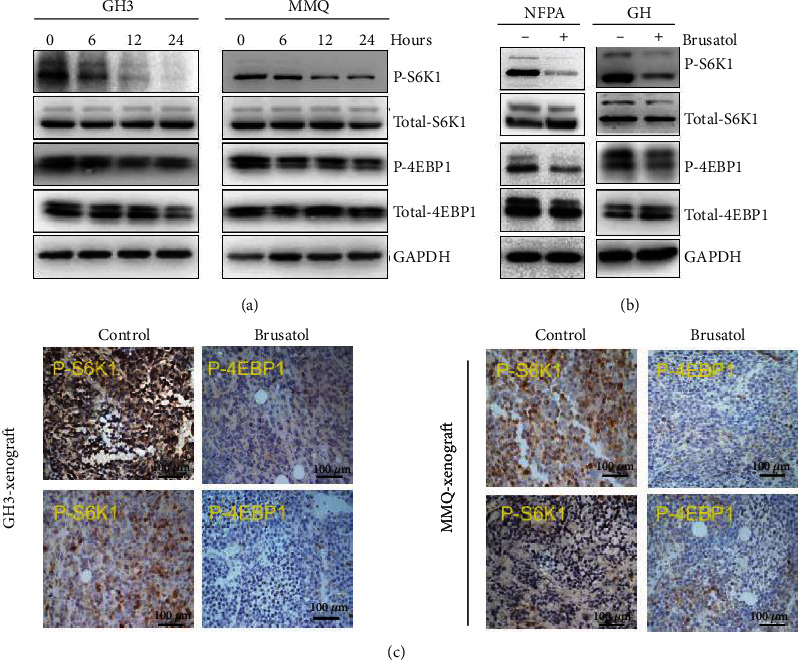
Brusatol (BT) downregulated the phosphorylation level of 4EBP1 and S6K1 in pituitary adenomas. (a) After 0–24 h treatment with BT (250 nM) in GH3 and MMQ cells, Western blot analysis was used to monitor the expression level of 4EBP1, S6K1, p-4EBP1, and p-S6K1. (b) Primary pituitary tumor cells (1 GHoma and 1 nonfunctional tumor) were treated with BT; Western blot analysis revealed the expression level of 4EBP1, S6K1, p-4EBP1, and p-S6K1. (c) Representative images of IHC-stained samples show that BT decreased p-4EBP1 and p-S6k1 expression in GH3 and MMQ xenograft models; scale bar, 100 *μ*m.

The effect of BT on the mTORC1 pathway in pituitary cells was investigated using immunoblotting. We observed that BT significantly inhibited mTORC1 signaling in a time-dependent manner in GH3 and MMQ cells, as indicated by decreased phosphorylation of S6K1 and 4EBP1, two key downstream effectors of the mTORC1 complex (Figure 4(a)). BT dramatically inhibited the phosphorylation of S6K1 and 4EBP1 in human nonfunctional pituitary adenoma and GH-secreting pituitary adenoma (Figure 4(b)). Consistent with the immunoblot results, BT significantly suppressed the expression of phosphorylated S6K1 (Figure 4(c)), suggesting that mTORC1 inhibition may contribute to BT-induced cell death in pituitary tumors.

### 3.5. ROS Play a Pivotal Role in BT-Mediated Cell Death and mTORC1 Inhibition

**Figure 5:**
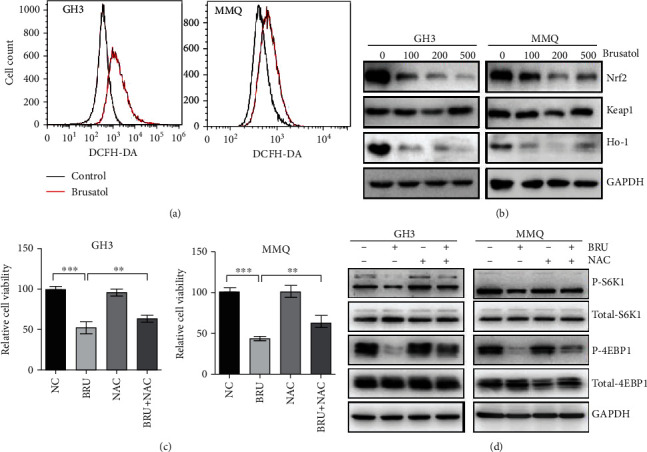
Brusatol (BT) increased ROS accumulation in GH3 and MMQ cells by inhibiting the Nrf2 pathway. (a) GH3 and MMQ cells treated with BT (250 nM) for 24 h were analyzed by flow cytometry after DCFH-DA staining to determine ROS levels. (b) GH3 and MMQ cells were treated with a range of concentrations of BT for 24 h. Western blot analysis showed that BT downregulated the expression of Nrf2 and its downstream gene Ho-1 but had no effect on Keap1. (c) GH3 and MMQ cell lines were treated with BT (250 nM) and/or NAC (100 *μ*M) for 24 h and cell viability measured by MTS assay. (d) GH3 and MMQ cell lines were treated with BT (250 nM) and/or NAC (100 *μ*M) for 24 h; Western blot analysis showed that NAC reversed the inhibitory effect of BT on inhibition of p-4EBP1 and p-S6K1. Data are presented as the mean ± SD. ^∗∗^*P* < 0.01; ^∗∗∗^*P* < 0.001.

We then investigated whether BT induces pituitary tumor cell death via ROS-mediated mTORC1 inhibition. We observed that BT treatment (250 nM) increased the ROS accumulation after 24 h in GH3 and MMQ cells (Figure 5(a)). Further, we tested the protein expression of Nrf2, Keap1, and Ho-1, which related to ROS accumulation, in GH3 and MMQ cells. BT decreased the expression of Nrf2 and Ho-1 but had no effect on Keap1 (Figure 5(b)). The ROS inhibitor N-acetylcysteine (NAC) reversed the BT-induced decrease in cell viability by 20.3% in GH3 cells and 21.8% in MMQ cells (Figure 5(c)) (*P* < 0.05). Western blot analysis revealed that BT inhibited the expression of p-S6K1 and p-4EBP1, and NAC abolished this decrease (Figure 5(d)). In summary, these results show that BT inhibits mTORC1 signaling and cell viability by increasing ROS levels as an upstream signal.

### 3.6. BT Augmented the Effects of Cabergoline in GH3 and MMQ Cells

**Figure 6:**
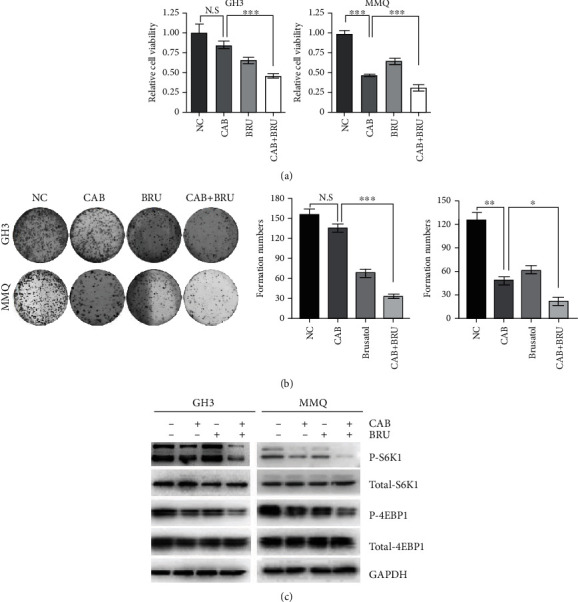
Brusatol (BT) increased the cytotoxicity of cabergoline in GH3 and MMQ cells. (a) GH3 and MMQ cells were treated with BT (250 nM) and/or cabergoline (50 *μ*M) for 24 h. Cell viability was measured by MTS assays, and (c) Western blot analysis was conducted to determine the level of p-4EBP1 and p-S6K1. (b) Representative images of colony formation assays. GH3 and MMQ cells were treated with BT (250 nM) and/or cabergoline (50 *μ*M) for 24 h. Colony formation was allowed to proceed for 14 days. Data are presented as the mean ± SD. ^∗^*P* < 0.05; ^∗∗^*P* < 0.01; ^∗∗∗^*P* < 0.001.

MTS and colony formation assays were used to determine whether BT potentiates the effects of cabergoline on cell viability and growth in GH3 and MMQ. Consistent with our previous studies, treatment with 50 *μ*M cabergoline had no effect on GH3 cells [22], but combined treatment with BT led to a significant reduction in cell viability of 54.1% (*P* < 0.001) (Figure 6(a)). Although 50 *μ*M cabergoline treatment alone effectively decreased MMQ cell proliferation, combined treatment with cabergoline and BT further reduced cell viability by 69.7% (*P* < 0.001) (Figure 6(b)). Similarly, combined treatment with cabergoline and BT led to evident inhibition of pituitary tumor cell growth.

To determine whether BT augments cabergoline-mediated inhibition of the mTORC1 pathway, we examined the expression of p-S6K1 and p-4EBP1 after combined treatment with cabergoline and BT. Western blot analysis showed greater decreases in p-S6K1 and p-4EBP1 expression in cells treated with cabergoline plus BT as compared to those treated with either drug alone (Figure 6(c)).

### 3.7. BT Augmented the Antitumor Activity of Cabergoline in GH3 Xenografts

**Figure 7:**
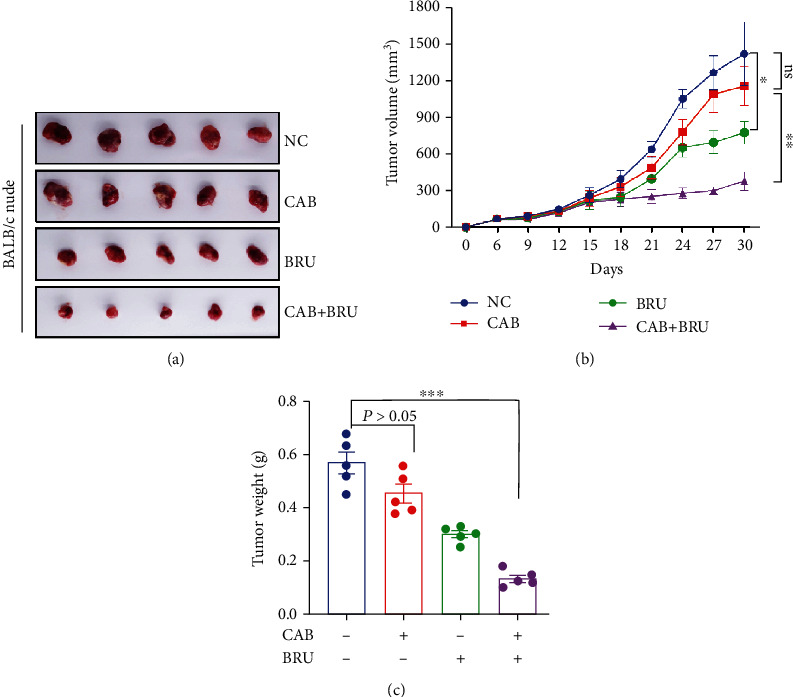
Brusatol (BT) augmented the cytotoxic effect of cabergoline in GH3 xenografts. (a) The 4 groups were treated with BT or/and cabergoline every other day. After 30 days of treatment, the nude mice were anesthetized, and the tumors were harvested. (b) Tumor volume was measured every 3 days (volume = *π*/6 × [larger diameter] × [smaller diameter]^2^), and the volume curve was determined using GraphPad Prism 8.0. (c) Comparison of the average xenograft tumor weight. Data are represented as the mean ± SD. ^∗^*P* < 0.05; ^∗∗∗^*P* < 0.001.

The effects of BT and cabergoline alone or in combination were investigated in GH3 cells subcutaneously implanted into nude mice. A clear difference in tumor size was observed between the cabergoline/BT combined treatment and the control or single-drug treatments (Figures 7(a) and 7(b)). The decrease in tumor weight was greater in those treated with cabergoline/BT combined than in controls (0.452 ± 0.079 g vs. 0.132 ± 0.032 g [*n* = 5]; *P* < 0.05) (Figure 7(c)).

These *in vitro* and *in vivo* data indicate that BT induces cell death by ROS-mediated inhibition of mTORC1 signaling in pituitary tumors and augments the antitumor activity of cabergoline.

## 4. Discussion

We observed that in GH3 and MMQ cells and several human pituitary adenoma primary cultures, BT inhibited cell growth and promoted apoptotic cell death *in vitro* and *in vivo*. Furthermore, combined treatment with BT and CAB increased tumor suppression *in vivo* and resulted in tumor-suppressive effects of CAB at a lower concentration. Our investigation of the mechanism underlying these effects showed that BT decreases mTORC1 signaling pathway activation by increasing ROS levels in pituitary tumor cells.

In the past two decades, DRD2 agonists such as CAB and bromocriptine (BRC) have been the first-choice treatment for controlling hyperprolactinemia and shrinking the volume of prolactinomas [11, 23]. However, due to drug resistance and lack of efficacy against other subtypes of pituitary tumors, new drugs are needed to treat pituitary adenomas or improve the efficacy of cabergoline. A previous study reports that artesunate and BRC have synergistic anticancer effects in pituitary adenoma [22], suggesting that traditional Chinese medicine extracts may have potential for treating pituitary tumors. BT is proven to inhibit the growth and ameliorate chemoresistance in a variety of cancer cells. Here, we report for the first time that BT inhibits pituitary tumor cell proliferation and growth. Using primary cultures of different pituitary tumor types, we observed that BT successfully suppressed cell growth in most pituitary tumor samples. BT is reported to increase ROS production by inhibiting NRF2 inhibition, thereby inducing ROS-mediated inhibition of cancer cell growth [6]. Our results indicate that BT inhibits pituitary cell growth by increasing the generation of ROS and that NAC reverses the effects of BT in GH3 and MMQ cells. Thus, we propose that BT induces ROS-mediated cell death in pituitary adenoma. This interesting finding warrants further exploration of the precise mechanism underlying this effect.

Recent investigations have revealed that the PI3K-AKT-mTOR pathway plays a pivotal role in tumorigenesis and chemoresistance [15, 24] in a variety of cancers, including pituitary tumors [25]. Our previous studies have shown that cabergoline inhibits activation of the AKT-mTOR pathway and induces autophagic cell death [20]. ROS-mediated AKT-mTOR inactivation is known to cause autophagic cell death in pituitary tumors [20, 26]. Ye et.al reported the involvement of mTOR inhibition in the BT-induced inhibition of cell proliferation and growth in hepatocellular carcinoma cells [8], but the underlying mechanism was unclear. Studies have shown that the phosphorylation of 4EBP1 and S6K1 is directly related to protein translation needed for tumor cell growth [27–29]. Interestingly, several studies have found that BT inhibits protein synthesis and promotes chemoresistance in cancer cells [30–32]. Our study shows that BT induces ROS accumulation and inhibits the phosphorylation of 4EBP1 and S6K1, the substrates of the mTORC1 complex. Furthermore, NAC significantly reversed the effect of BT in GH3 and MMQ cells. These results suggest that BT-induced ROS inhibit mTORC1 activation and may inhibit protein synthesis in pituitary tumors. However, further exploration is needed in follow-up studies to confirm this potential mechanism as underlying the effects of BT.

We reported previously that chloroquine, a clinical antimalarial drug, increases the effectiveness of cabergoline in several subtypes of pituitary tumors [33]. Thus, combining cabergoline with other drugs may be an effective strategy for addressing drug resistance in prolactinomas. BT is proven to ameliorate chemoresistance in multiple types of cancer cells, including breast cancer [34], hepatoma cells [35], non-small-cell lung cancer cells [36], squamous cell carcinoma [37], and pancreatic cancer cells [38]. Here, we have shown that BT inhibits the phosphorylation of 4EBP1 and S6K1, leading to increased efficacy of cabergoline in GH3 and MMQ cells. Consistent with our previous study [33], treatment with cabergoline (0.5 mg/kg every other day) had no effect on tumor growth in GH3 xenografts. However, combined treatment with cabergoline and BT clearly inhibited tumor growth in nude mice. While Western blot analysis showed no decrease in p-4EBP1 or p-S6K1 in cells treated with cabergoline alone, combined treatment with cabergoline and BT decreased p-4EBP1 and p-S6K1 levels. These findings strongly indicate that BT augments the anticancer effects of cabergoline by inhibiting the mTORC1 pathway in pituitary tumor cells.

## 5. Conclusion

In summary, our study reveals important inhibitory effects of BT on pituitary tumor growth and clearly demonstrates the benefit of combined treatment with cabergoline and BT for pituitary tumor suppression. These findings provide a rationale for conducting pilot clinical studies of this combination therapy for treating prolactinomas and other pituitary tumors.

## Data Availability

The data used to support the findings of this study are available from the corresponding author upon request.

## Supplementary Materials

Supplementary materials Patients' information of pituitary tumors which conducted in this study were listed in the Supplementary table.

## References

[1] Avila-Carrasco L., Majano P., Sánchez-Toméro J. A. (2019). Natural plants compounds as modulators of epithelial-to-mesenchymal transition. *Frontiers in pharmacology*.

[2] Cai S. J., Liu Y., Han S., Yang C. (2019). Brusatol, an NRF2 inhibitor for future cancer therapeutic. *Cell & Bioscience*.

[3] Zhou M.-M., Zhang W.-Y., Li R.-J. (2018). Anti-inflammatory activity of Khayandirobilide A from Khaya senegalensis via NF-kappa B, AP-1 and p 38 MAPK/Nrf 2/HO-1 signaling pathways in lipopolysaccharide-stimulated RAW 264.7 and BV-2 cells. *Phytomedicine*.

[4] Sun X., Wang Y., Ji K. (2020). NRF2 preserves genomic integrity by facilitating ATR activation and G2 cell cycle arrest. *Nucleic Acids Research*.

[5] Mata-Greenwood E., Cuendet M., Sher D., Gustin D., Stock W., Pezzuto J. M. (2002). Brusatol-mediated induction of leukemic cell differentiation and G(1) arrest is associated with down-regulation of c-myc. *Leukemia*.

[6] Ren D., Villeneuve N. F., Jiang T. (2011). Brusatol enhances the efficacy of chemotherapy by inhibiting the Nrf2-mediated defense mechanism. *Proceedings of the National Academy of Sciences of the United States of America*.

[7] Tang X., Fu X., Liu Y., Yu D., Cai S. J., Yang C. (2020). Blockade of glutathione metabolism in IDH1-mutated glioma. *Molecular cancer therapeutics.*.

[8] Ye R., Dai N., He Q. (2018). Comprehensive anti-tumor effect of Brusatol through inhibition of cell viability and promotion of apoptosis caused by autophagy via the PI3K/Akt/mTOR pathway in hepatocellular carcinoma. *Biomedicine & Pharmacotherapy*.

[9] Gillam M. P., Molitch M. E., Lombardi G., Colao A. (2006). Advances in the treatment of prolactinomas. *Endocrine reviews.*.

[10] Melmed S. (2020). Pituitary-tumor endocrinopathies. *The New England Journal of Medicine*.

[11] Huang H. Y., Lin S. J., Zhao W. G., Wu Z. B. (2018). Cabergoline versus bromocriptine for the treatment of giant prolactinomas: a quantitative and systematic review. *Metabolic Brain Disease*.

[12] Colao A., Savastano S. (2011). Medical treatment of prolactinomas. *Nature reviews Endocrinology.*.

[13] Olafsdottir A., Schlechte J. (2006). Management of resistant prolactinomas. *Nature clinical practice Endocrinology & metabolism.*.

[14] Molitch M. E. (2003). Dopamine resistance of prolactinomas. *Pituitary*.

[15] Saxton R. A., Sabatini D. M. (2017). mTOR signaling in growth, metabolism, and disease. *Cell*.

[16] Mossmann D., Park S., Hall M. N. (2018). mTOR signalling and cellular metabolism are mutual determinants in cancer. *Nature Reviews. Cancer*.

[17] Caron A., Briscoe D. M., Richard D., Laplante M. (2018). DEPTOR at the nexus of cancer, metabolism, and immunity. *Physiological Reviews*.

[18] Yao H., Tang H., Zhang Y. (2019). DEPTOR inhibits cell proliferation and confers sensitivity to dopamine agonist in pituitary adenoma. *Cancer Letters*.

[19] Guo S., Zhang J., Wei C. (2020). Anticancer effects of brusatol in nasopharyngeal carcinoma through suppression of the Akt/mTOR signaling pathway. *Cancer Chemotherapy and Pharmacology*.

[20] Leng Z. G., Lin S. J., Wu Z. R. (2017). Activation of DRD5 (dopamine receptor D5) inhibits tumor growth by autophagic cell death. *Autophagy*.

[21] Lyublinskaya O. G., Ivanova J. S., Pugovkina N. A. (2017). Redox environment in stem and differentiated cells: a quantitative approach. *Redox Biology*.

[22] Wang X., Du Q., Mao Z. (2017). Combined treatment with artesunate and bromocriptine has synergistic anticancer effects in pituitary adenoma cell lines. *Oncotarget*.

[23] Bevan J. S., Webster J., Burke C. W., Scanlon M. F. (1992). Dopamine agonists and pituitary tumor shrinkage. *Endocrine Reviews*.

[24] Hausch F., Kozany C., Theodoropoulou M., Fabian A. K. (2013). FKBPs and the Akt/mTOR pathway. *Cell Cycle*.

[25] Chen R., Duan J., Li L. (2017). mTOR promotes pituitary tumor development through activation of PTTG1. *Oncogene*.

[26] Lin S. J., Leng Z. G., Guo Y. H. (2015). Suppression of mTOR pathway and induction of autophagy-dependent cell death by cabergoline. *Oncotarget*.

[27] Holz M. K., Ballif B. A., Gygi S. P., Blenis J. (2005). mTOR and S6K1 mediate assembly of the translation preinitiation complex through dynamic protein interchange and ordered phosphorylation events. *Cell*.

[28] Avdulov S., Li S., Van Michalek D. B. (2004). Activation of translation complex eIF4F is essential for the genesis and maintenance of the malignant phenotype in human mammary epithelial cells. *Cancer Cell*.

[29] She Q. B., Halilovic E., Ye Q. (2010). 4E-BP1 is a key effector of the oncogenic activation of the AKT and ERK signaling pathways that integrates their function in tumors. *Cancer Cell*.

[30] Harder B., Tian W., La Clair J. J. (2017). Brusatol overcomes chemoresistance through inhibition of protein translation. *Molecular Carcinogenesis*.

[31] Turpaev K., Krizhanovskii C., Wang X., Sargsyan E., Bergsten P., Welsh N. (2019). The protein synthesis inhibitor brusatol normalizes high-fat diet-induced glucose intolerance in male C57BL/6 mice: role of translation factor eIF5A hypusination. *The FASEB Journal*.

[32] Oh E. T., Kim C. W., Kim H. G., Lee J. S., Park H. J. (2017). Brusatol-mediated inhibition of c-Myc increases HIF-1alpha degradation and causes cell death in colorectal cancer under hypoxia. *Theranostics.*.

[33] Lin S. J., Wu Z. R., Cao L. (2017). Pituitary tumor suppression by combination of cabergoline and chloroquine. *The Journal of Clinical Endocrinology and Metabolism*.

[34] Olayanju A., Copple I. M., Bryan H. K. (2015). Brusatol provokes a rapid and transient inhibition of Nrf2 signaling and sensitizes mammalian cells to chemical toxicity-implications for therapeutic targeting of Nrf2. *Free radical biology & medicine.*.

[35] Lee J. H., Mohan C. D., Deivasigamani A. (2020). Brusatol suppresses STAT3-driven metastasis by downregulating epithelial-mesenchymal transition in hepatocellular carcinoma. *Journal of Advanced Research*.

[36] Liu P., Wu D., Duan J. (2020). NRF2 regulates the sensitivity of human NSCLC cells to cystine deprivation-induced ferroptosis via FOCAD-FAK signaling pathway. *Redox Biology*.

[37] Lee J. H., Rangappa S., Mohan C. D. (2019). Brusatol, a Nrf2 inhibitor targets STAT3 signaling cascade in head and neck squamous cell carcinoma. *Biomolecules*.

[38] Xiang Y., Ye W., Huang C. (2018). Brusatol enhances the chemotherapy efficacy of gemcitabine in pancreatic cancer via the Nrf2 signalling pathway. *Oxidative medicine and cellular longevity.*.

