# Supplementary material for: Brusatol Inhibits Tumor Growth and Increases the Efficacy of Cabergoline against Pituitary Adenomas
Source: Oxid Med Cell Longev. 2021 Jun 16;2021:6696015. doi: 10.1155/2021/6696015 (PMC8221873; doi:10.1155/2021/6696015)
Supplement: Supplementary materials — Patients' information of pituitary tumors which conducted in this study were listed in the Supplementary table. [file 6696015.f1.pdf]

Patients' information of pituitary tumors

Patient Number	Gender	Age	Tumor type	Tumor size (mm)	Knosp Grade	Prolactin Levels (ng/ml)	GH level(ng/ml)
1	M	45	NFPA	40*36*26	Ⅲ	8.39	0.06
2	F	48	NFPA	40*20*20	Ⅳ	1.37	0.33
3	M	41	NFPA	23*22*18	Ⅰ	5.92	0.05
4	M	72	NFPA	20*25*14	Ⅱ	6.69	0.25
5	F	40	GHoma	19*11*13	Ⅱ	15.62	17.56
6	F	22	PRLoma	26*22*20	Ⅱ	387.01	0.65

F: Femal; M: Male; NFPA: non-functional pituitary adenoma; GHoma: growth hormone secreting pituitary adenoma; PRLoma: Prolactinoma;
